# ﻿Progress or burden? Formal description of every apparently new species available in collections is neither necessary nor useful

**DOI:** 10.3897/zookeys.1214.130592

**Published:** 2024-10-01

**Authors:** Bernhard A. Huber, Hubert Szymański, Alice Bennett-West

**Affiliations:** 1 Zoological Research Museum Alexander Koenig, LIB, Bonn, Germany Zoological Research Museum Alexander Koenig Bonn Germany; 2 Włocławek, Poland Unaffiliated Włocławek Poland; 3 Clevedon, Somerset, UK Unaffiliated Clevedon United Kingdom

**Keywords:** Alien species, Europe, new species, open nomenclature, Pholcidae, *Quamtana*, single-sex description, South Africa, species lists, taxonomy

## Abstract

A new species of the Sub-Saharan spider genus *Quamtana* Huber, 2003 is described that has been collected in garden centers in Poland and the UK. Its closest known relative is probably *Q.lotzi* Huber, 2003, known from Free State Province in South Africa. Working on the premise that placing species in time and space is the fundamental task of taxonomy, and acknowledging that we cannot provide biologically meaningful spatial information for this species, we prefer open nomenclature to make this species known to science without formally describing it, using the unique provisional name *Quamtana* sp. ZFMK Ar 24490 aff.lotzi. We argue that the judicious use of open nomenclature can serve to improve the quality of species lists, reducing the noise in large-scale analyses of biodiversity data. We expand this argument to ‘fragmentary’ species descriptions in general, such as single-sex descriptions in large genera with many male-only and female-only descriptions. Not every taxonomic act allowed by the Code is necessarily beneficial. Under certain conditions, the informal description of a putatively new species may serve science better than a formal description based on inadequate material or data.

## ﻿Introduction

Species are often, and in many contexts, conveniently divided into two categories, the known and the unknown (Stork 1997; Reaka-Kudla 2001; Costello and Wilson 2010; Costello 2015; Huber and Chao 2019; Moura and Jetz 2021; Liu et al. 2022). This is not only an overly simplistic view of our knowledge of the earth’s species diversity (Lawton 1993; Loxdale et al. 2016; Meier et al. 2022) but also a potentially misleading one. ‘Known’, i.e. formally described, species include a wide and continuous spectrum of taxa that range from well-studied model organisms to those of which we know little else than their names, and those whose names should not even exist because they are synonyms. Informed ignorance is a powerful research tool (Hortal et al. 2015), but in taxonomy, the level of ignorance hidden behind a scientific name is often not easily accessible except by a few specialists. Taxonomists are well aware of the fact that focusing on formally undescribed taxa is only part of the job. In fact, more time and effort are often required to give meaning to names of species that are formally described but basically unknown than to formally describing a new species (e.g., Riedel et al. 2013; Kehlmaier et al 2019).

To some degree, this is a legacy of 250 years of taxonomic history, and of the rules (Codes) that the taxonomic community has chosen to adopt. These rules happen to conserve taxonomic decisions as long as certain formal criteria are followed, irrespective of scientific merit. However, the problem is not only historical. New species are still massively being formally described based on suboptimal or inadequate material or data. Countless species are being described from a single specimen, a single sex, or a single locality; from poorly preserved specimens; from photographs rather than physical specimens; from specimens without precise locality data; from specimens without any biologically meaningful type locality; etc. (e.g., Lim et al. 2012; Santos et al. 2016; Deng et al. 2019). From a theoretical point of view, there is nothing fundamentally wrong with this; this is how science works. Hypotheses (in this case, species) are proposed and further research can refine or reject these hypotheses. Every species description is necessarily incomplete in some respect, and what constitutes a reasonably ‘complete’ description varies with the taxonomic group, time, the needs of the end user, available research infrastructure, etc. (Durkin et al. 2020; Sharkey et al. 2021a, b). In addition, ‘fragmentary’ descriptions obviously can and usually do, provide useful information. Single-sex descriptions, for example, do not compromise the locality data nor the morphological, molecular, natural history etc. data provided about the known sex.

However, formal descriptions based on suboptimal or inadequate material or data can be harmful by introducing problems that interfere with scientific progress. For example, single-sex descriptions may become problematic if many male-only and female-only species are proposed within a taxon of superficially similar species; species with imprecise locality data may become problematic if the rough coordinates given in databases are used in biogeographic analyses; conservation efforts and ecological studies may be misled by problematic taxonomic decisions hidden behind the authority of a scientific name (Bortolus 2008; Maldonado et al. 2015; Ely et al. 2017; Nekola and Horsák 2022). At some point and under certain circumstances, the potential problems of a formal description may outweigh its advantages.

Our example to illustrate this point is a new species of daddy long-legs spider (Pholcidae) of the genus *Quamtana* Huber, 2003 that was recently discovered in several European garden centers. *Quamtana* is a Sub-Saharan genus, with a particularly high diversity in South Africa (Huber 2003). A few species have been found further north, reaching Central and East Africa. *Quamtana* spiders have been introduced to Europe before: two species were reported from German plant markets and greenhouses where they seemed to have established viable populations (Huber et al. 2015). Obviously, all these species have been introduced from Africa, probably with plants. They are not indigenous European species, and their natural distributions are unknown. Taxonomy is fundamentally about placing species in time and space, but in the case of *Quamtana* spiders in Europe, we lack biologically meaningful information on the spatial component. Our aim here is not to argue that formally describing such species is necessarily bad; it is obviously permitted by the relevant taxonomic Code (ICZN 1999). Instead, we favor the approach of ‘open nomenclature’ (Bengtson 1988; Sigovini et al. 2016; Minelli 2019; Horton et al. 2021) for cases such as this, and claim that (1) all the relevant information available at this point can be provided without formally naming the species, and (2) informal descriptions of ‘problematic’ species helps improve the quality of formal species lists and databases.

## ﻿Materials and methods

This study is primarily based on the examination of specimens deposited in Zoologisches Forschungsmuseum Alexander Koenig, Bonn, Germany (ZFMK). Further material is deposited in the private collections of the second and third authors. The taxonomic description follows the style of the only available revision of the genus (Huber 2003; based on Huber 2000). Measurements were done on a dissecting microscope with an ocular grid and are in mm unless otherwise noted; eye measurements are +/- 5 µm. Photos were made with a Canon EOS 2000D digital camera mounted on a Nikon SMZ18 stereo microscope or a Nikon Coolpix 995 digital camera mounted on a Leitz Dialux 20 compound microscope. CombineZP (https://combinezp.software.informer.com/) was used for stacking photos. Drawings are partly based on photos that were traced on a light table and later improved under a dissecting microscope, or they were directly drawn with a Leitz Dialux 20 compound microscope using a drawing tube. The cleared epigynum was stained with chlorazol black. Abbreviations used in figures only are explained in the figure legends. Abbreviations used in the text: **ALE** = anterior lateral eye(s); **AME** = anterior median eye(s); **a.s.l.** = above sea level; L/d = length/diameter; **PME** = posterior median eye(s).

## ﻿Results

**Order Araneae Clerck, 1757**

**Family Pholcidae C.L. Koch, 1850**

**Genus *Quamtana* Huber, 2003**

### ﻿*Quamtana* sp. ZFMK Ar 24490 aff.lotzi

Figs 1–5

**Figure 1.**
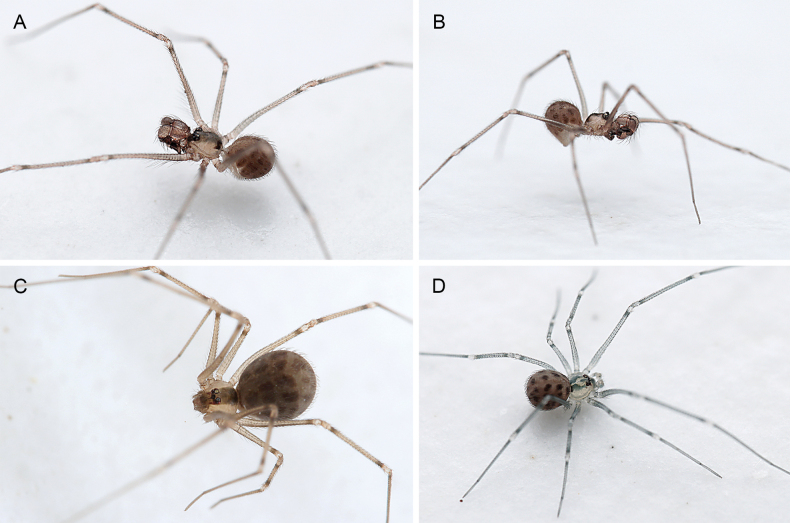
*Quamtana* sp. ZFMK Ar 24490 aff.lotzi; live specimens, from Poland, Bydgoszcz **A, B** male **C** female **D** juvenile (penultimate instar male).

**Figure 2.**
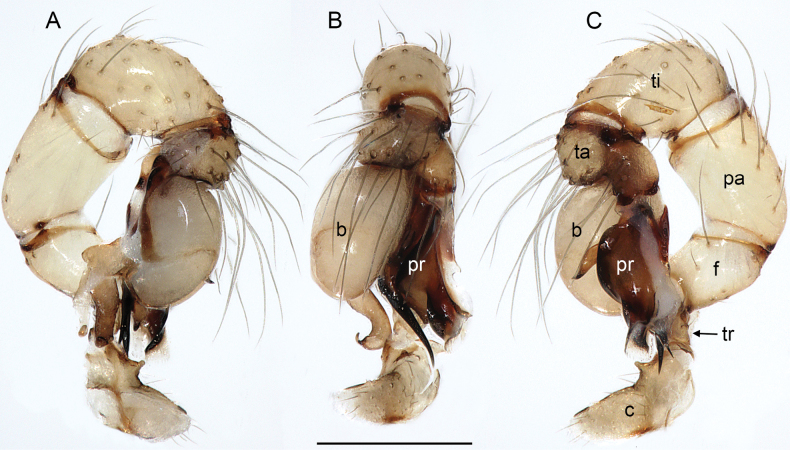
*Quamtana* sp. ZFMK Ar 24490 aff.lotzi; male from Poland, Bydgoszcz, ZFMK Ar 24490. Left palp, prolateral (**A**), dorsal (**B**), and retrolateral (**C**) views. Abbreviations: b, genital bulb; c, coxa; f, femur; pa, patella; pr, procursus; ta, tarsus; ti, tibia; tr, trochanter. Scale bar: 0.3 mm.

**Figure 3.**
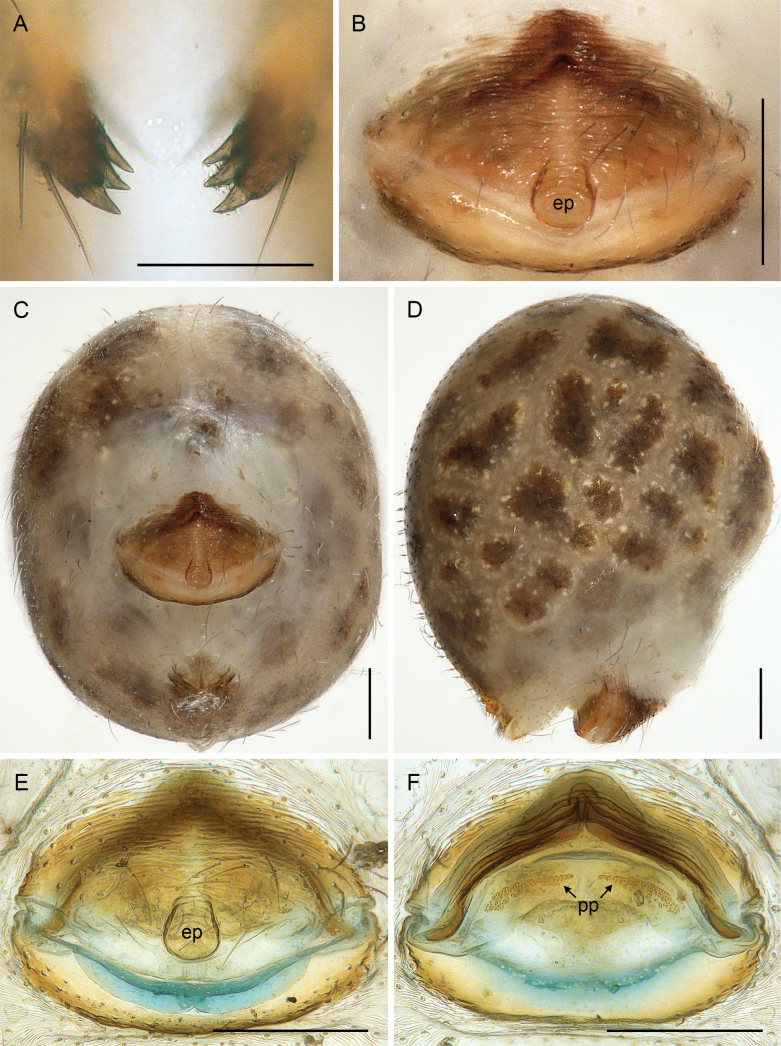
*Quamtana* sp. ZFMK Ar 24490 aff.lotzi; male and female from Poland, Bydgoszcz, ZFMK Ar 24490 **A** frontal male cheliceral apophyses, frontal view **B** epigynum, ventral view **C, D** female abdomen, ventral and lateral views **D, E** cleared female genitalia, ventral and dorsal views. Abbreviations: ep, epigynal pocket; pp, pore plates. Scale bars: 0.05 mm (**A**); 0.2 mm (**B–F**).

**Figure 4.**
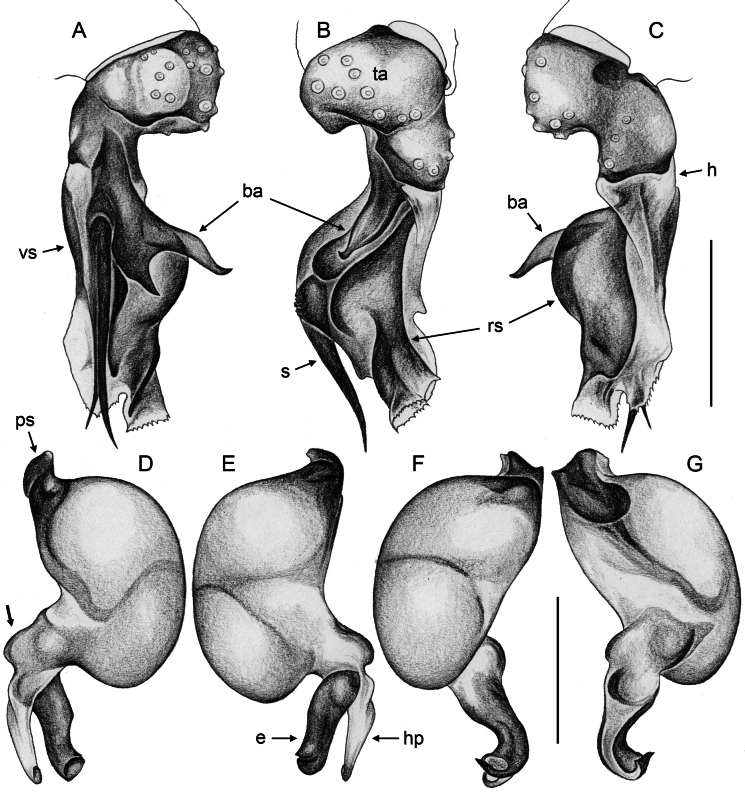
*Quamtana* sp. ZFMK Ar 24490 aff.lotzi; male from Poland, Bydgoszcz **A–C** left palpal tarsus and procursus, prolateral, dorsal, and retrolateral views, ZFMK Ar 24490 **D–G** left genital bulb, prolateral, retrolateral, dorsal, and ventral views, ZFMK G161; bold arrow in **D** points at ventral protrusion proximally on bulbal processes. Abbreviations: ba, bifid apophysis; e, putative embolus; h, hinge between proximal and distal parts of procursus; hp, hooked bulbal process; ps, proximal bulbal sclerite; rs, retrolateral-dorsal sclerite; s, hinged spine; ta, tarsus; vs, ventral sclerite. Scale bars: 0.2 mm.

**Diagnosis.** Small, long-legged pholcid (Fig. 1) with eight eyes and with median projection on male clypeus. Distinguished from most known congeners (except *Q.lotzi* Huber, 2003) by longer than wide male palpal patella (Fig. 2), by presence of distinct apophysis on male palpal coxa (Fig. 2), by presence of three rather than two modified hairs on each male cheliceral apophysis (Fig. 3A), and by distinctive ventral protrusion proximally on bulbal processes (bold arrow in Fig. 4D). From *Q.lotzi* and other congeners also by details of procursus shape (Fig. 4A–C; bifid prolateral-dorsal apophysis, long and slender prolateral-ventral spine; large rounded retrolateral-dorsal sclerite) and by details of genital bulb shape (Fig. 4D–G; two processes originating from common basis, one sclerotized, possibly containing sperm duct, other largely membranous, hooked at tip). Female distinguished from known congeners by large median epigynal pocket (Fig. 3B, E) rather than pair of pockets; female of *Q.lotzi* unknown.

**Material examined.** Poland – **Kuyavia-Pomerania** • 1 ♂, 1 ♀ abdomen, 2 juvs; Bydgoszcz; 53.123°N, 18.064°E; 50 m a.s.l.; in OBI market; 20 Jan. 2024; H. Szymańksi leg.; ZFMK Ar 24490 • 1 ♂, 1 ♀ (abdomen transferred to ZFMK Ar 24490), in pure ethanol; same collection data as for preceding; ZFMK G161 • 1 ♂, 2 ♀, 6 juvs; Toruń; 53.024°N, 18.670°E; 65 m a.s.l.; in OBI market; 2 Mar. 2024; H. Szymański, D. Szymański, D. Szymański leg.; in private collection H. Szymańksi. Great Britain – **England** • 2 ♂; Almondsbury, garden center; 51.550°N, 2.577°W; 6 Nov. 2023; A. Bennett-West leg.; ZFMK Ar 24652 • 2 ♀; same collection data as for preceding; in private collection A. Bennett-West.

**Description. Male** (ZFMK Ar 24490). ***Measurements*.** Total body length 1.5, carapace width 0.54. Distance PME-PME 80 µm; diameter PME 75 µm; distance PME-ALE 20 µm; distance AME-AME 20 µm; diameter AME 35 µm. Leg 1 missing; tibia 2: 1.25, tibia 3: 0.75, tibia 4: 1.23.

***Colour*** (in ethanol). Prosoma and legs mostly pale ochre, carapace with dark median mark including ocular area and clypeus; sternum monochromous; legs with darker rings on femora (subdistally) and tibiae (proximally and subdistally); abdomen pale gray, with dark internal marks dorsally and laterally, ventrally with indistinct rectangular darker mark in front of gonopore.

***Body*.** Habitus as in Fig. 1A, B. Ocular area moderately raised. Carapace without thoracic groove. Clypeus with distinct median process ~60 µm long, ending in two tines. Sternum wider than long (0.42/0.38), unmodified. Abdomen globular, conical at spinnerets.

**Figure 5.**
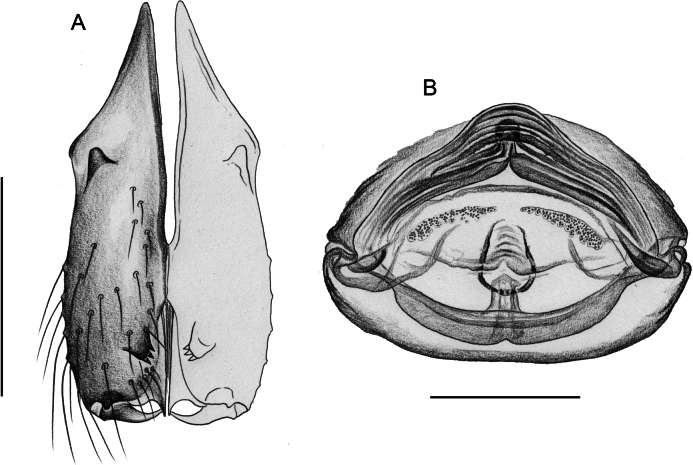
*Quamtana* sp. ZFMK Ar 24490 aff.lotzi; male and female from Poland, Bydgoszcz **A** male chelicerae, frontal view, ZFMK Ar 24490 **B** cleared female genitalia, dorsal view, ZFMK G161. Scale bars: 0.2 mm.

***Chelicerae*.** As in Fig. 5A; with pair of proximal lateral processes weakly sclerotized and directed proximally and pair of distal apophyses close to median line, each with three modified (conical) hairs (Fig. 3A); without stridulatory files.

***Palps*.** As in Fig. 2; coxa with distinct ventral apophysis; trochanter with short retrolateral process (~40 µm long); femur short, distally widening but otherwise unmodified; femur-patella joints slightly shifted toward prolateral side; patella very long; tibia-tarsus joints shifted toward retrolateral side; procursus (Fig. 4A–C) consisting of short proximal part and complex distal part hinged against proximal part; proximal part on prolateral-dorsal side with bifid apophysis with long hinged spine; distal part with large retrolateral-dorsal sclerite connected ventrally and prolaterally with membranous structures and simple flat ventral sclerite; genital bulb (Fig. 4D–G) with strong proximal sclerite and two distal processes, one sclerotized (putative embolus), the other one semitransparent and distally hooked; with ventral protrusion proximally on bulbal processes (bold arrow in Fig. 4D).

***Legs*.** Without spines, without curved hairs, without sexually dimorphic short vertical hairs. Ventral hairs proximally on femora long, in particular on femur 2 (~350 µm long, proximal half of femur).

***Supplementary information on other males***: ZFMK G161: leg 1 length: 9.00 (2.40 + 0.25 + 2.45 + 3.10 + 0.80), tibia 2 missing, tibia 3: 0.98, tibia 4: 1.45; tibia 1 L/d: 41; retrolateral trichobothrium of tibia 1 at 11%; prolateral trichobothrium absent on tibia 1. Ventral hairs on femur 1 also long, but only in proximal fifth of femur, and shorter than on femur 2 (~300 µm); tarsus 1 with ~15 pseudosegments, distally distinct.

ZFMK Ar 24652: tibia 1: 2.75.

**Female.** In general, very similar to male (Fig. 1C) but clypeus unmodified, ventral hairs on leg femora shorter (up to ~150 µm). Tibia 1 missing. Epigynum (Fig. 3B–D) protruding, anterior plate triangular, with distinct median pocket in posterior position; posterior epigynal plate wide and simple. Internal genitalia (Fig. 3E, F) with anterior arc and pair of long pore plates in transversal position.

**Natural history.** In the garden centers in Poland, the specimens were found in sections with xerophilic plants such as cacti and succulents, under bark, under stones and bricks, and under flowerpots. In the garden center in England, the specimens were found on the underside of shelving, also close to xerophilic plants and succulents.

**Relationships.** The present species shares with *Q.lotzi* a number of characters that are unique in *Quamtana*: (1) long male palpal patella (Fig. 2); (2) presence of distinct apophysis on male palpal coxa (Fig. 2); (3) presence of three rather than two modified hairs on each male cheliceral apophysis (Fig. 3A); and (4) distinctive ventral protrusion proximally on bulbal processes (bold arrow in Fig. 4D). We suspect that most or all of these similarities are derived and that the two species are sister taxa.

**Distribution and geographic origin.** The species has so far only been found in garden centers in Poland and England. The plants in the Polish stores originate from several parts of the world, and they reach Poland via a nursery in the Netherlands. It is thus not possible to trace the spiders back via the plants. For England, we do not have any information regarding the origin of plants where specimens were found. The only clue regarding the origin we have is thus the putative sister species, *Q.lotzi*. This species is known from a single male specimen originating from the Koppiesdam Nature Reserve (27°13'S, 27°42'E; Huber 2003) in Free State, South Africa. We thus suspect that the present species may also originate from approximately this area.

**Remark.** The format of the provisional name above follows the suggestions in Horton et al. (2021). The two species of *Quamtana* previously reported from Germany (Huber et al. 2015) were not named according to such criteria that include unique registration numbers and facilitate integration into databases. We thus propose the following names for those two taxa: for “*Quamtana* sp. A” we propose “*Quamtana* sp. ZFMK Ar 12707 aff. kabale”, for “*Quamtana* sp. B” we propose “*Quamtana* sp. ZFMK Ar 12704 aff.mabusai”.

## ﻿Discussion

Species represent a core unit of life, and taxonomy strives to discover, delimit, describe and name species according to scientific standards. Species names can be viewed as hypotheses that follow from the discovery, delimitation and description process; unambiguous names are essential tools to communicate knowledge about the world’s biodiversity across countries, time and scientific disciplines. Species (names) that are aggregated into species lists and databases affect views and decisions far beyond biological disciplines such as evolutionary and ecological research. They have potential impact on conservation, trade, agriculture, development, species invasions and health (Thomson et al. 2018; Garnett et al. 2020; Hobern et al. 2021; Sandall et al. 2023).

Taxonomic hypotheses, like hypotheses in any other field of science, range from poorly supported to well supported. Taxonomists are mostly well aware of which taxa in their specific group of interest are problematic and which can be regarded as solid and less likely to be rejected by further research. However, this information, even if published, is not visible in formal species names and is usually not visible in species lists. Instead, species lists usually contain every taxon name that meets the relevant criteria of the respective Code. While researchers in other biological disciplines can safely ignore published information that is arguably unreliable, formal taxonomic names are stubborn: once created they are here to stay, both individually and in lists, until they are formally rejected (Agatha et al. 2021).

Recent heated debates among taxonomists reflect this dilemma: while some argue that taxonomy has to follow new approaches to massively speed up species descriptions, others feel confronted with a mass of species they consider poorly supported but that they feel forced to include in species lists and to take into account in subsequent research (e.g., Sharkey et al. 2021a, 2021b, 2022 vs. Ahrens et al. 2021; Zamani et al. 2022a, b; Meier et al. 2022). A similar debate has been going on about species descriptions based on photographs, without physical vouchers (reviewed in Kasalo et al. 2021). A less controversial yet pervasive problem is synonyms. Depending on the taxonomic group, 20% and more of the species names in lists may be synonyms (Solow et al. 1995; Dubois 2008). Synonyms have been and are being created for a number of reasons. Some potential synonyms are indeed hard to avoid or to solve, for example, those resulting from different species concepts or delimitation criteria. Others appear avoidable. For example, genera with large numbers and percentages of male-only and female-only descriptions are likely to include synonyms. While the arachnological community, as an example, has long ago realized that describing new species based on juvenile specimens creates more problems than it solves, single sex descriptions continue to be produced massively. In the Asian funnel weaver genus *Draconarius* Ovtchinnikov, 1999, 155 of the currently 270 nominal species are known from a single sex (WSC 2024). Of these 155 single-sex descriptions (108 female-only, 47 male-only), 131 have been published since 2000. *Draconarius* is no exception; several other large spider genera have even higher numbers and higher percentages of male-only and female-only descriptions (WSC 2024). Taxonomists producing such descriptions are of course aware of the problem and correctly point out that it can be solved by further research. Until then, however, all these species will appear in lists and databases, with potential impacts far beyond the narrow niche of alpha taxonomy.

Informal species descriptions make knowledge available while keeping species lists and databases of formally described species as clean as possible. The idea of ‘open nomenclature’ is, of course, not new, and early proponents have argued that it should be seen as an essential tool in the taxonomist’s repertoire (e.g., Bengtson 1988). Not only is it a compromise between formal description and retention of information, but its “careful and judicious use … reflects scientific honesty” (Bengtson 1988). Recent proponents have emphasized the potential of open nomenclature, provided that its use becomes standardized and made compatible with modern biodiversity research (Sigovini et al. 2016; Minelli 2019; Horton et al. 2021). The basic motivation is certainly widely shared: to maximize efforts to prevent the publication of insufficient descriptions and redescriptions, and to improve the reliability of biological datasets and their utility in informing policy and management (Agatha et al. 2021; Horton et al. 2021).

Ryberg and Nilsson (2018) claimed that “an inflation in names that are difficult to interpret or apply may hamper taxonomy more than an inflation in species descriptions without valid names.” What if Sharkey et al. (2021a) had published their tremendous amount of valuable data on wasp diversity in Costa Rica without producing more than 400 formal species names? What if proponents of species descriptions based on photographs instead of physical vouchers had published their discoveries of putative new species without binomials (as suggested by Löbl et al. 2016; Horton et al. 2021; and implemented in Kasalo et al. 2021)? What if taxonomists working on *Draconarius* spiders had produced formal single-sex descriptions for only one sex, while informally providing all the relevant information on ‘species’ known from the other sex only? For pholcid spiders, the first author has formally described over 900 species, but none are based on females only. Knowing one particular sex in all species (males in the case of Pholcidae, because they generally are easier to diagnose) minimizes the problem of creating different names for male-only and female-only ‘species’ when they are in fact the same species. In Pholcidae, apparent female-only species were not ignored but described informally (e.g., in Huber et al. 2023a, b). For a slightly different approach in dealing with the problem of matching sexes in spiders, also involving informal descriptions of putative new species, see Izquierdo and Ramírez (2017). Apart from keeping species lists clean, this may motivate future collectors to search for the missing sex and formally describe the species if, in fact, new.

It could be argued that informal names are not appropriately considered or entirely invisible in catalogs, species lists and databases and are consequently neglected in further research, legislation, conservation efforts, etc. However, whether this is a problem of open nomenclature or a problem of the species lists, catalogs, and legislation is open to debate. The WSC (2024) for example, includes a reference to the formally undescribed species of *Quamtana* from Germany mentioned above, duly separate from the list of formally described species, while other informally described species (e.g. those in Huber et al. 2023a, b; and those in Izquierdo and Ramírez 2017) are ignored. There is obviously not only a need for consistency but mainly for a way to incorporate available information into species lists and databases that allows the end user to decide which information is valuable for a particular purpose and which is not.

In sum, we work on the premise that placing species in time and space is the fundamental task of taxonomy, but we acknowledge that a fragmentary description (e.g., one without a meaningful space component, as in our case) can carry valuable information. We thus use open nomenclature to make this species known to science without formally describing it. All users of taxonomy might profit from a careful case-by-case evaluation by taxonomists of whether the available data justify formal species description or not. We extend this argument to all species descriptions based on fragmentary data, as for example, on single-sex descriptions in species-rich taxa that already have many male-only and female-only species. Formal description of every putatively new species available in collections is not necessarily beneficial and potentially even harmful.

## ﻿Additional information

### Author ORCIDs

Bernhard A. Huber https://orcid.org/0000-0002-7566-5424

Hubert Szymański https://orcid.org/0000-0003-0549-8912

### Data availability

All of the data that support the findings of this study are available in the main text.
